# Further pathogenicity testing of *Verticillium nonalfalfae*, a biocontrol agent against the invasive Tree of Heaven (*Ailanthus altissima*), on non-target tree species in Europe

**DOI:** 10.1007/s12600-022-01032-z

**Published:** 2022-11-05

**Authors:** Yvonne Lechner, Oliver Maschek, Thomas Kirisits, Erhard Halmschlager

**Affiliations:** Department of Forest and Soil Sciences, Institute of Forest Entomology, Forest Pathology and Forest Protection (IFFF), University of Natural Resources and Life Sciences, Vienna (BOKU), Peter-Jordan-Str. 82 (Franz-Schwackhöfer-Haus), 1190 Vienna, Austria

**Keywords:** Tree of Heaven, *Verticillium nonalfalfae*, Host range, Biological control, Non-target-effects, Susceptibility

## Abstract

*Verticillium nonalfalfae* is an effective biological control agent against the highly invasive Tree of Heaven (*Ailanthus altissima*) in Europe, and previous research on ten economically and ecologically important tree species occurring in Austria have so far not revealed undesired non-target effects. In this study, another nine tree species including five native, two non-native as well as two invasive alien tree species were tested for susceptibility to the particular strain of *V*. *nonalfalfae* (Vert56) used for biological control of *A. altissima*. Stem inoculations on potted seedlings revealed that this strain of *V*. *nonalfalfae* is generally host-adapted to *A. altissima*. It induced chlorosis, necrosis and wilting already within two weeks post inoculation on *A. altissima* and resulted in almost completely defoliated or dead seedlings at the end of the vegetation period. Apart from two species (*Quercus rubra* and *Sorbus aucuparia*), that suffered from other abiotic/biotic agents, no mortality was observed on all other tree species tested; however, symptoms caused by other abiotic factors were also found on *Prunus avium* and *Ulmus glabra*. All tested tree species exhibited vascular discolorations and the fungus could be re-isolated at varying frequencies (6—100%) from inoculated seedlings of all non-target tree species, although five of these species exhibited no external symptoms. Results confirmed high susceptibility (S) of *A. altissima* to *V.* *nonalfalfae*, whereas *Acer platanoides*, *Castanea sativa*, *Q. rubra*, *S. aucuparia* and *U. glabra* were considered as tolerant (T), and *A. negundo, P. avium*, *P. serotina* and *Q. petraea* were rated as possible resistant (PR) due to the low rates of re-isolation.

**Supplementary Information:**

The online version contains supplementary material available at 10.1007/s12600-022-01032-z.

## Introduction

*Ailanthus altissima* (thereafter mostly referred to just as *Ailanthus*), native to China and North Vietnam, is a highly invasive alien tree species in Europe and North America (Hu, 1979; Kowarik & Säumel, 2014). Since its first introduction into Europe about 250 years ago, *Ailanthus* has evolved from a rare exotic species to a widespread neophyte. It has been spread by humans on all continents except Antarctica and was subsequently cultivated for many different uses (e.g. as ornamental tree, for erosion control or as food for honeybees; Kowarik & Säumel, 2007, 2014). After the Second World War, *Ailanthus* became established on sites, which were opened up by bombs as well as on ruins and fallow land, where it benefits from its ecological characteristics as a pioneer species (Gutte et al., 1987; Kowarik & Böcker, 1984; Kowarik & Säumel, 2007). Nowadays, *Ailanthus* is mainly found in areas with a temperate or Mediterranean climate all over the world. In Mediterranean areas *Ailanthus* is widespread, whereas in Central Europe it is largely confined to lowlands, low mountain ranges or cities (Kowarik & Säumel, 2014), which is due to the more favorable climatic conditions (e.g. urban heat islands) at these sites (Gutte et al., 1987; Kowarik & Säumel, 2014; Punz et al., 1998). In urban habitats *Ailanthus* is commonly found on built-up areas and fallow land, in crevices of sidewalks, along fence rows, stonewalls, roadsides, railroad lines, waterways, electricity line routes but also on industrial wastelands and green spaces (Kowarik & Säumel, 2007). In addition to urban habitats, *Ailanthus* also occurs in near-natural floodplains, dry forests or grasslands and on rocky sites, and is also able to invade borders of meadows, agricultural fields and vineyards, which has become increasingly relevant within the last decade (Kowarik & Böcker, 1984; Gutte et al., 1987; Kowarik & Säumel, 2007; Kowarik, 2010; Dauth & Halmschlager, pers. comm).

The following characteristics facilitate the establishment of *Ailanthus*: (I) It is very tolerant to various climatic conditions and soil properties (Kowarik & Böcker, 1984; Kowarik & Säumel, 2007). (II) It has a rapid youth growth (Halmschlager & Maschek, 2019), a high capacity of generative (Wickert et al., 2017) and vegetative reproduction (Bory et al., 1991; Kowarik & Säumel, 2007) (rapid and early fructification and the ability to produce numerous root suckers and stump sprouts) and is (III) quite resistant to various pollutants, drought and salt (Filippou et al., 2014). (IV) In addition, it has allelopathic properties (Gómez-Aparicio & Canham, 2008; Heisey, 1997; Lin et al., 1995), and therefore it is able to displace native species and endangers natural ecosystems. (V) Furthermore, there are hardly any natural enemies outside of its natural range (Ding et al., 2006; Hu, 1979; Kowarik & Böcker, 1984; Kowarik & Säumel, 2007; Siegrist & Holdenrieder, 2016).

As *Ailanthus* is thermophilic, global warming will further favor its spread, as with rising temperatures it will occupy significantly larger areas by the middle of the 21^st^ century (Kleinbauer et al., 2010). *Ailanthus* is therefore considered as a problematic species (Kowarik & Säumel, 2014), which is on the list of the 20 most invasive alien plants in Europe (Siegrist & Holdenrieder, 2016) and, since 2019, also on the official EU list of invasive alien species (European Commission, 2019).

In Austria, *Ailanthus* is – besides *Acer negundo* (Boxelder) – a major problem in riparian forests in floodplains along the Danube (Drescher & Magnes, 2002; Gutte et al., 1987; Ließ & Drescher, 2008), where it is one of the most aggressive and highly invasive neophytes. Many different methods to control *Ailanthus* have been tested so far: mechanical treatment (e.g. cut stump/double cut stump, girdling/partial girdling or grubbing; Constán-Nava et al., 2010; Ließ, 2007; Müller, 2012) of *Ailanthus* is expensive, often unsuccessful and can even have the opposite effect (i.e. development of numerous root suckers and stump sprouts), whereas chemical control (i.e. stem drilling and application of glyphosate, imazapyr or triclopyr; DiTomaso & Kyser, 2007; Ließ, 2007; Lewis & McCarthy, 2008; Badalamenti et al., 2015) or a combined mechanical/chemical method (i.e. cut stump or girdling with glyphosate application treatment; Badalamenti & La Mantia, 2013; Constán-Nava et al., 2010; Ließ, 2007; Müller, 2012) is ecologically not worthwhile or even forbidden in some regions, such as in water protection areas, near-natural ecosystems, biosphere reserves or national parks.

The above-mentioned limitations of mechanical and chemical control methods prompted the idea of searching for an effective biological control agent of *Ailanthus* (Halmschlager & Maschek, 2019). Subsequently, the wilt pathogen *Verticillium* *nonalfalfae* (at that time still identified as *Verticillium* *albo*-*atrum*) could be isolated in 2011 from a dying *Ailanthus* tree in southern Styria (Austria) for the first time in Europe (Maschek & Halmschlager, 2016a). *V*. *nonalfalfae* was described by Inderbitzin et al. (2011) as part of taxonomic changes within the genus *Verticillium.* Based on molecular data, the authors concluded that *V.* *albo‐atrum* s.l., which has been treated as a single species so far, actually comprises the three morphologically very similar species *V.* *albo‐atrum* s.s., *Verticillium alfalfae* and *V*. *nonalfalfae*. Thus, all previous morphology-based host range records referring to *V*. *albo atrum* s.l. have to be taken with care, because without molecular data or reference cultures, identifications can no longer be clearly related to one of the three taxa in this species complex (Inderbitzin & Subbarao, 2014; Inderbitzin et al., 2011).

Following its initial isolation from *Ailanthus* in 2011 in Austria (Maschek & Halmschlager, 2016a), *V*. *nonalfalfae* turned out to be a suitable candidate as biological control agent against *Ailanthus* (Maschek & Halmschlager, 2017, 2018). Pathogenicity and high virulence of the fungus towards *Ailanthus* was also found in three provinces of the USA, namely in Pennsylvania (Schall & Davis, 2009a), Virginia (Snyder et al., 2013) and Ohio (Rebbeck et al., 2013), and recently also in another European country, in Spain (Moragrega et al., 2021). However, host specificity testing of a potential biological control agent is required in order to evaluate the possible risk of unintentional side-effects to non-target plant species within the envisaged area of release. Previous surveys of co-occurring woody plants in the field around naturally or artificially infected *Ailantus* trees as well as results from artificial inoculation trials suggested host adaptation of selected *V*. *nonalfalfae* strains/isolates obtained from *Ailanthus* to the host they had initially been isolated from (Schall & Davis, 2009a, b; Kasson et al., 2014, 2015; O'Neal & Davis, 2015a, b; Maschek & Halmschlager, 2018; Moragrega et al., 2021). Nevertheless, there are strains of *V*. *nonalfalfae* that might cause economical losses on agricultural crops such as tomato, cucumber, spinach, alfalfa as well as on some cultivars of potato and hop (Inderbitzin & Subbarao, 2014; Jing et al., 2018; Svara et al., 2019). However, results from a recent study revealed that some of those crops rated as highly susceptible in the literature are not susceptible to *V. nonalfalfae* isolate Vert56 used for biological control of *Ailanthus* in Europe (Dauth et al., 2022).

Results of host range studies by Schall and Davis (2009b), Kasson et al. (2015) and Maschek and Halmschlager (2018) suggest that *V*. *nonalfalfae* has a much narrower host range compared to *V*. *albo‐atrum* s.l.: in the USA, natural infections of co-occurring forest tree species by *V*. *nonalfalfae* were only observed in two of 38 examined species [devil´s walking stick (*Aralia spinosa*) and striped maple (*Acer pensylvanicum*)] growing in stands with declining *Ailanthus*, whereas in inoculation studies varying levels of wilt and mortality were found in ten out of 71 tested non-target woody species (Kasson et al., 2015). Host range tests by Maschek and Halmschlager (2018) on potted seedlings of eight indigenous and two invasive alien tree species that grew intermingled with dead and dying *Ailanthus* in Austria and/or were reported as susceptible to *V*. *albo atrum* s.l. revealed no susceptibility of any of the tested tree species, although vascular discolorations developed in all inoculated species.

Based on this previous study (Maschek & Halmschlager, 2018), we tested another nine non-target tree species, all of which are considered to be susceptible to *Verticillium* spp. according to the literature (Butin, 2019; Kasson et al., 2015; Pegg & Brady, 2002; Sinclair & Lyon, 2005), for their susceptibility to the potential biocontrol agent of *Ailanthus* after artificial inoculation. Thus, the aim of the present study was to gain further knowledge on the potential host range of *V*. *nonalfalfae* isolate Vert56 of strain G1/5, the particular strain used for biological control of *Ailanthus*, in order to exclude undesirable effects on non-target tree species. Based on the observed external symptoms (leaf chlorosis, necrotic leaf margins, wilting and loss of leaves, dieback and mortality), the presence or absence of vascular discolorations in the stem and the results of re-isolations from inoculated potted seedlings, the tested species were classified as susceptible, tolerant, possibly resistant or resistant to *V*. *nonalfalfae*.

## Material and methods

### Study site and experimental plants

The study was carried out from summer 2017 to spring 2018 and repeated for two of the tested tree species from June 2019 to October 2020 on a fenced, outdoor test area at the University of Natural Resources and Life Sciences, Vienna (BOKU). The stem inoculation experiment was conducted on potted seedlings of *Acer* *negundo*, *A*. *platanoides*, *A*. *altissima* (which served as control to demonstrate pathogenicity and virulence of the applied *V. nonalfalfae* isolate), *Castanea sativa*, *Prunus avium*, *P*. *serotina*, *Quercus petraea*, *Q*. *rubra*, *Sorbus aucuparia* and *Ulmus glabra* (Table 1). Bare rooted seedlings (size category 120–150 cm) of *C*. *sativa*, *P*. *serotina*, *Q*. *petraea*, *Q*. *rubra* and *U*. *glabra* were obtained in May 2017 from the tree nursery “Murauer” (Hübing, Upper Austria), and those of *A*. *platanoides*, *P*. *avium* and *S*. *aucuparia* from the tree nursery “Schwanzer” (Langenschönbichl, Lower Austria). Five-year-old potted *A*. *negundo* and *Ailanthus* seedlings were already available at the start of the experiment. The seedlings from nurseries were potted into 5.5-l plastic pots in a ready mixed substrate (Terra Vita Pflanzensubstrat, T6-Gehölze/Stauden; Franz Kranzinger GmbH, Straßwalchen, Austria) and fertilized with 20 g Osmocote® Exact Hi.End 8-9 M (15–9-11 + 2MgO + TE) per pot. Due to the size of some plants, a radical root cut was necessary in some species (e.g. *Q. rubra and S.* *aucuparia*), in order to fit root balls into the 5.5-l plastic pots.

**Table 1:** Results of the artificial inoculations of seedlings of nine non-target tree species and *Ailanthus altissima* with *Verticillium nonalfalfae* isolate Vert56 carried out in 2017 and in 2019 (re-isolations were made in 2018 and for the repeated experiment with *Q. rubra* and *S.* *aucuparia* also in 2020)

Species	Number of seedlings Inoculated/controls (n)		External disease symptoms (%)		Mortality (%)		Vascular discolorations (%)		Successful re-isolations total (%)		Status
	2017	2019	2017	2019	2017	2020	2018	2020	2018	2020	
*Ailanthus altissima*	19/4	-	100	-	42	-	100	-	70^b^	-	S
*Acer negundo*	18/4	-	0	-	0	-	89	-	44	-	PR
*Acer platanoides*^#^	19/4	-	0	-	0	-	74	-	100	-	T
*Castanea sativa*	18/4	-	0	-	0	-	44	-	61	-	T
*Prunus avium*^#^	22/4	-	27^a^	-	0	-	91	-	9	-	PR
*Prunus serotina*	18/4	-	0	-	0	-	89	-	6	-	PR
*Quercus petraea*^#^	21/4	-	0	-	0	-	62	-	33	-	PR
*Quercus rubra*	21/4	16/4	38^a^	19^a^	0	19^a^	67	100	24	88	PR/T
*Sorbus aucuparia*^#^	22/4	15/4	55^a^	73^a^	14^a^	13^a^	77	87	73	87	T/T
*Ulmus glabra*^#^	22/4	-	5^a^	-	0	-	95	-	64	-	T

*S* susceptible (wilt/mortality, high rate of re-isolation), *T* tolerant (limited/no wilt or mortality, medium or high rate of re-isolation), *PR* possibly resistant (no wilt/mortality, low rate of re-isolation), *R* resistant (no wilt/mortality, no re-isolation)

^#^ Tree species native to Austria

^a^ Symptoms/mortality attributed to other abiotic/biotic agents

^b^ In the case of *Ailanthus* re-isolations were carried out only from seedlings that still exhibited at least some living stem/root tissue (7 out of 10); seedlings which already had dead wood as well as seedlings with bark necrosis and/or bark degradation/discolouration were excluded

All seedlings of a tree species were labelled with same-coloured ribbons (using different colours for each tree species) and each seedling was additionally marked with a number. Seedlings were subjected to ambient conditions, watered on demand with tap water and treated with non-systemic pesticides (Netzschwefel Mehltau-Pilzfrei; Scotts CELAFLOR Handelsgesellschaft mbH and Neudosan AF Neu Blattlausfrei; W. Neudorff GmbH KG) against powdery mildew and aphids, as necessary.

Due to the ambiguous results obtained for *Q. rubra* and *S. aucuparia* in 2018, the experiment was repeated with these two species in 2019/20. To reduce the assumed negative impact of root cuts in the previous experiment, already potted plants of the size category 50–80 cm were ordered and potted in late spring 2019 again in 5.5-l plastic pots, using the same substrate as described above.

### Inoculation

Pure colonies of *V*. *nonalfalfae* isolate Vert56 of strain G1/5 were grown for three weeks on 2% malt extract agar [MEA; 20 g DiaMalt malt extract (Hefe Schweiz AG, Stettfurt, Switzerland), 16 g Becoagar agar (W. Behrens & Co, Hamburg, Germany), 1000 ml tap water] amended with 100 mg streptomycin sulphate (SM; Calbiochem, Merck KGaA, Darmstadt, Germany) added after autoclaving. For inoculum preparation, cultures of *V*. *nonalfalfae* were flooded with sterile water and conidia were carefully released with a sterile glass spatula. The resulting conidial suspension was carefully mixed, adjusted to 1 × 10^7^ conidia ml^−1^, kept at 4 °C and used within 3 days. A germination test conducted immediately prior to inoculations confirmed that viability of conidia exceeded 80%.

Stem inoculations of seedlings were conducted between July 13^th^—17^th^, 2017, and for the additional *Q. rubra* and *S. aucuparia* seedlings again on June 6^th^, 2019 using the inoculation method described by Maschek and Halmschlager (2016b), which allows the quick, reliable and accurate application of a defined volume of liquid inoculum into the sapwood of lignified plants. Inoculations were performed on two opposite positions at different heights of each seedling to prevent partial girdling of seedlings: one point was located at about 5 cm above ground level, and the other one at the opposite side, approximately 10 cm above the first one. Each seedling was wounded at these points, using a sterile straight gouge (Stubai® woodcarving tool Sweep 7, size 6–8 mm, depending on the stem diameter) and allowing the seedling to absorb 2 × 0.5 ml of the inoculum (i.e. conidial suspension or sterile water for the controls). Apart from the seedlings listed in Table 1 (comprising 15 – 22 inoculated seedlings and 4 controls for each species), a few (2–3) *Ailanthus* seedlings were inoculated on each inoculation date in order to confirm effectivity of the inoculum.

As *V*. *nonalfalfae* is known to be sensitive to high temperatures, the maximum and the mean daily temperatures were recorded during the whole observation period in 2017 using a datalogger.

### Monitoring of disease symptoms

Disease severity (DS) was monitored biweekly until the end of the vegetation period in 2017 and – due to COVID-19 restrictions – only twice in 2020 for the repeated experiment with *Q. rubra* and *S. aucuparia*, using a slightly adapted classification system used by Bejarano-Alcázar et al. (1996), Schall and Davis (2009a, b) and Maschek and Halmschlager (2018) that ensures comparability with previous scientific studies. This ordinal rating system uses values from 0 to 4 to classify disease severity: 0 = no symptoms/healthy leaves, 1 = 1–33% foliage affected/chlorotic leaves, 2 = 34–66% foliage affected/necrotic leaf margins, 3 = 67–99% foliage affected/wilting leaves, 4 = totally defoliated or dead seedlings (Fig. S1, supplemental material). In some case where the classification did not fit ordinal rating due to reasons described below, we divided the classes 0 to 3 into three additional subclasses x.25, x.50 or x.75 with x = 0–3) to count for individuals that did not fit the ordinal rating scale. For instance, a seedling that was totally defoliated but had developed some water sprouts was rated with 3.75 instead of 4.

Obtained disease severity (DS) ratings were used to determine the mean disease severity (MDS) for every assessment date by calculating the arithmetic mean. Resulting values were displayed as trend line with MDS on the y-axis and weeks post inoculation (WPI) on the x-axis. The disease progress was analysed using the area under the disease-progress curve (AUDPC) using the formula AUDPC = ∑ [(y_i_ + y_i+1_)/2)][t_i+1_–t_i_] where y_i_ is the disease severity rating, t_i_ is the time of the _i_th rating and i = 0, 1, 2…n–1 (Jeger & Viljanen-Rollinson, 2001).

### Statistical analyses

Since data were normally distributed, the obtained AUDPC values were subjected to a two-factor analysis of variance (ANOVA) with the factors "variant of treatment" (inoculated or control) and "tree species". Post-hoc Scheffé test was applied to detect significant differences between inoculated seedlings of *A. altissima*, *Q. rubra* and *S.* *aucuparia*, whereas differences between treatments of the same species were analysed using Welch´s test. For all tests the level of significance was set at α = 0.05. All analyses were conducted using the statistical software IBM SPSS Statistics for Windows, Version 24.0 (IBM Corp. Released 2016. Armonk, NY). No statistical analyses were carried out for those tree species for which treatment with *V*. *nonalfalfae* showed no “effect” (i.e. when the obtained AUDPC values of the inoculated and the control group where overlapping almost identically on the x-axis), which was the case for the species *A*. *negundo*, *A*. *platanoides*, *C*. *sativa*, *P*. *serotina*, *Q*. *petraea* and *U*. *glabra*.

### Vascular discolorations in sapling stems and re-isolation

**Fig. 1:**
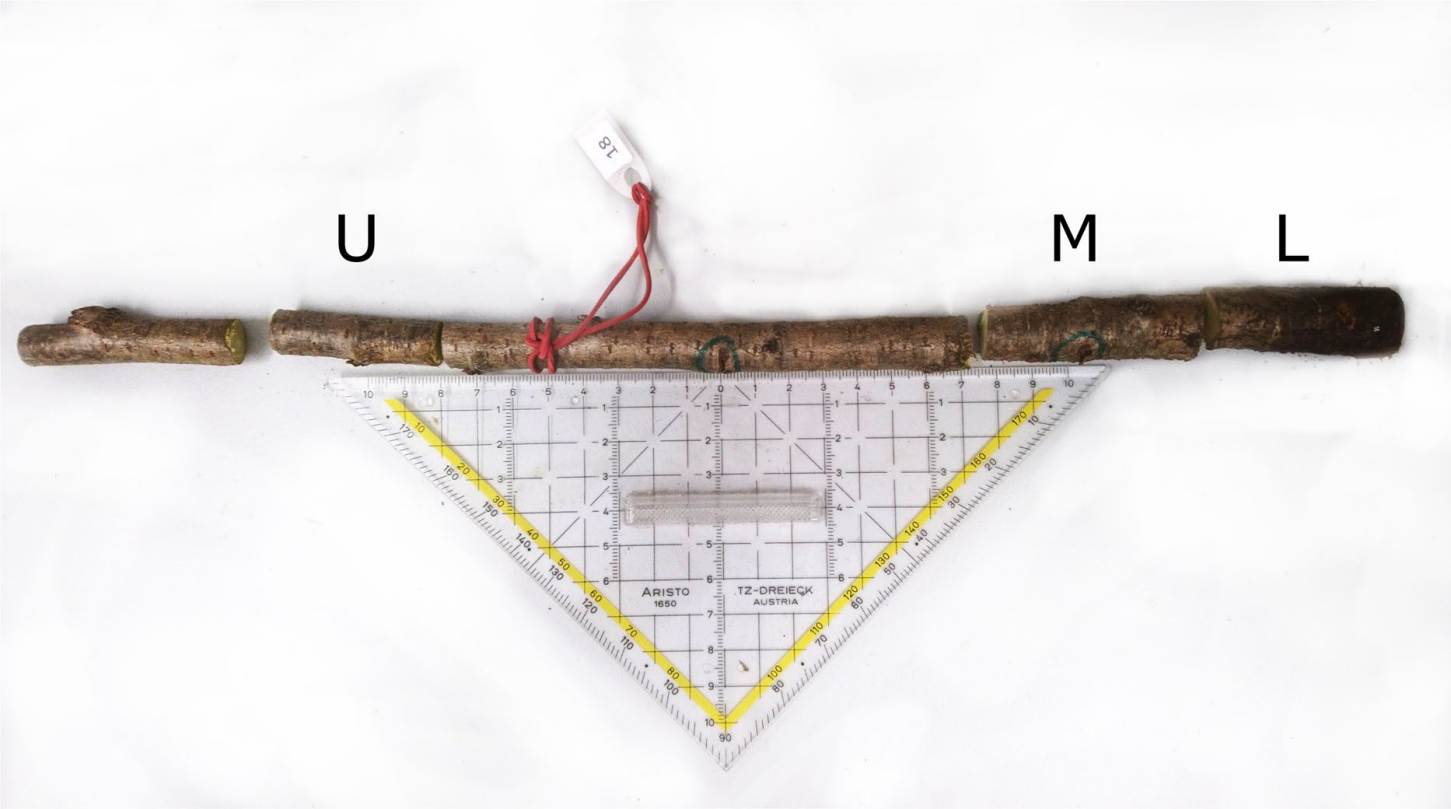
Stem sections used for re-isolations: U = upper 5-cm sample, M = middle 5-cm-sample containing the ground-level inoculation site, L = lower 5-cm sample; the blue rings indicate the two points of inoculation being approximately 10 cm apart from each other (the upper inoculation point was turned about-face to be visible)

Upon termination of the experiment in March 2018 (35 WPI) and in October 2020 (70 WPI) for the repeated experiment, all inoculated seedlings including controls were cut at ground level and a 35-cm-long section comprising both (lower and upper) inoculation sites was excised from seedling stems and transported immediately to the laboratory. There, three 5-cm-long samples were cut out of each 35-cm stem section using sterile pruning shears: (1) the lower sample was located 5 cm below the ground-level inoculation site, (2) the middle 5-cm-sample contained the ground-level inoculation site and (3) the upper sample comprised the part of the stem which was about 10 cm above the upper inoculation site (Fig. 1).

Samples were surface-sterilized using 96% ethanol (1 min), rinsed afterwards with sterile water (30 s) and were then split longitudinally under sterile conditions. Thereafter, samples were inspected for vascular discolorations characteristic for *Verticillium* infections (Butin, 2019; Sinclair & Lyon, 2005) on both cross and longitudinal sections. Representative vascular discolorations in cross and longitudinal section of the sapwood from each tree species were photographed.

For re-isolation, four tissue samples were excised from vascular discolorations or from the transition zone between living and necrotic sapwood using a sterilized scalpel. Tissue samples were plated on MEA + SM. Each Petri dish was labelled with a code for the tree species, tree number and re-isolation position. Petri dishes were kept in the dark at about 22 °C for nine days (Domsch et al., 1980) and the resulting cultures were examined for *V*. *nonalfalfae*. According to Maschek and Halmschlager (2018) re-isolations were considered as successful if *V*. *nonalfalfae* could be re-isolated from at least one of the three sampled sections obtained from one seedling.

Based on the observed disease severity and the rate of re-isolation the tested species were finally classified as susceptible (S), tolerant (T), possible resistant (PR) or resistant (R) to *V*. *nonalfalfae* isolate Vert56 (see legend of Table 1 for details).

## Results

**Fig. 2:**
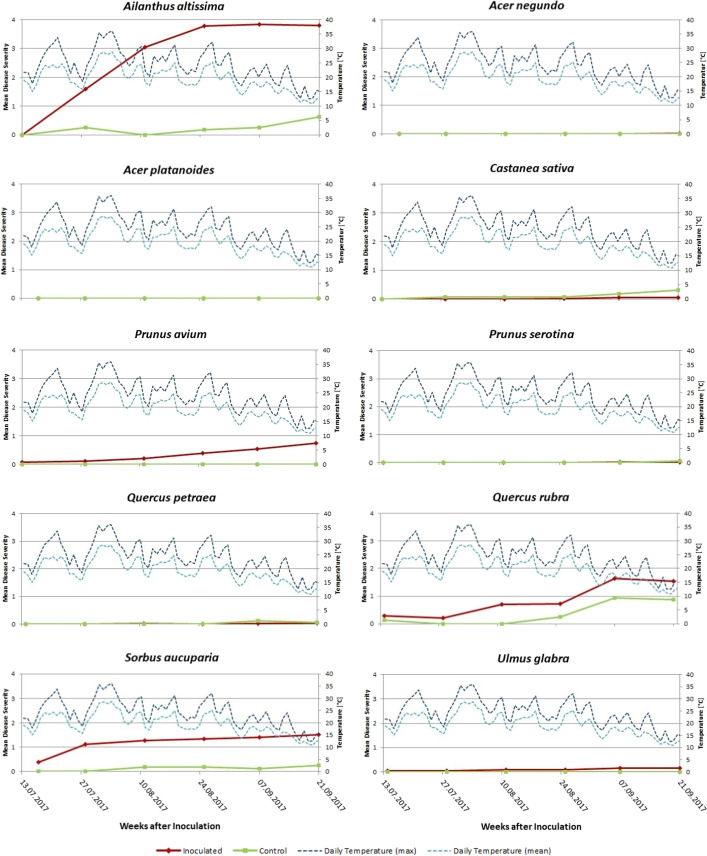
Disease progression curves showing the mean disease severity for inoculated seedlings (red curves) and controls (green curves) *of Ailanthus altissima* and the tested 9 non-target tree species. The green and red dots indicate the dates of evaluation. Dashed lines indicate the maximum (dark blue) and the mean daily temperature (light blue) during the observation period

During the observation period, recorded maximum daily temperatures on the test area exceeded 30 °C on 13 days and 35 °C on 3 days (31.07, 02.08 and 03.08.2017), whereas mean daily temperatures were in a range that should be optimal or at least suitable for growth of *V*. *nonalfalfae* (Fig. 2)*.*

### External disease symptoms and seedling mortality

Stem-inoculated *Ailanthus* seedlings showed a rapid disease progression on 100% (19 of 19 plants) of the trees: Already 2 weeks post-inoculation (WPI) 58% (11 of 19 trees) of the treated *Ailanthus* plants exhibited minor to severe disease symptoms (mean disease severity [MDS] of 1.59), such as chlorotic, necrotic or/and wilting leaves as well as defoliation (Fig. 2). Two weeks later, all inoculated seedlings already exhibited symptoms of a verticilliose and MDS had increased to 3.04. MDS culminated 8 WPI reaching a MDS of 3.8 and stayed constant until at the end of the vegetation period. Mortality occurred on 8 of 19 trees (42%), with the remaining 58% showing a MDS of 3.66. *Ailanthus* seedlings of the control group neither developed leaf necrosis nor wilt symptoms or dieback, but all trees showed a few chlorotic leaves and/or missing leaflets (Table 1). Thus, differences in AUDPC values between inoculated *Ailanthus* seedlings and controls were highly significant (p < 0.0001).

No symptoms were detected in either the inoculated or the control group of seedlings of *A*. *negundo* and *A*. *platanoides*.

Seedlings of *C*. *sativa* also did not develop any symptoms until 6 WPI. However, 8 WPI 22% of the inoculated seedlings (4 of 18) but also 75% of the controls (3 of 4) developed minimal leaf margin necrosis, though no wilting symptoms were recorded. At the end of the vegetation period in 2017, MDS of the inoculated *Castanea* seedlings had reached 0.04, whereas it was 0.31 on the controls (Fig. 2).

Five seedlings of *P*. *avium* already showed minor symptoms at time of inoculation, resulting in a MDS of 0.07. Two WPI mean disease severity of the inoculated seedlings was 0.11, which increased to 0.74 at the end of the vegetation period (Fig. 2). Overall, 27% (6 of 22) of the inoculated seedlings had developed symptoms such as leaf margin necrosis and curled or dropped leaves, whereas no symptoms were detected on the remaining seedlings. In addition, UV damage was also observed on those inoculated seedlings. In contrast, controls did not develop any symptoms during the entire evaluation period.

Inoculated and control seedlings of *P*. *serotina* hardly developed any disease symptoms during the whole vegetation period in 2017. However, 8 WPI one inoculated seedling and 10 WPI one inoculated as well as one control plant exhibited very slight leaf margin necrosis and were therefore classified with a disease severity of 0.25, resulting in a MDS of 0.01 and 0.06, respectively (Fig. 2).

Test plants (treated seedlings and controls) of *Q*. *petraea* also did not develop any symptoms related to an infection with *Verticillium*. However, 8 WPI two seedlings of the control group developed minor symptoms, which had disappeared by the end of the experiment.

Some seedlings of *Q*. *rubra* (inoculated plants as well as controls) already had symptoms (reduced leaf mass, leaf margin necroses) before inoculation, resulting in a MDS of 0.3 (inoculated) and 0.13 (controls) on the day of inoculation. In both groups the MDS decreased 2 WPI to a MDS of 0.21 (inoculated) and of 0 (controls), respectively. Thereafter, a slight increase of disease severity was observed on the inoculated seedlings, which stagnated until 6 WPI. However, 8 WPI disease symptoms again increased on the inoculated seedlings and at the end of the vegetation period MDS had reached 1.54 (Fig. 2). Although none of the inoculated test plants died, a considerable portion (8 of 21; 38%) developed symptoms. Control trees were at first inconspicuous, but from 6 WPI onwards, one individual was classified with 1 (= 1–33% foliage affected/chlorotic leaves), and symptoms identical to those on the inoculated seedlings increased until 8 WPI on that individual plant (Fig. S2, supplementary material). This point is also supported by the statistical analyses, which revealed no significant differences (p = 0.227) of AUDPC levels between the treated and the control group, whereas significantly lower AUDPC levels (p < 0.0001) were obtained for inoculated *Q*. *rubra* seedlings compared to those of *A. altissima*. In the repeated experiment in 2019/20, the MDS of inoculated *Q*. *rubra* seedlings was 0.59 at the first evaluation date (13.07.2020) and 0.75 at the termination of the experiment (08.10.2020). Development of disease symptoms followed by mortality was only observed on 19% (3 of 16) of the inoculated seedlings, whereas all other inoculated seedlings as well as all controls did not show any symptoms on both evaluation dates.

On *S*. *aucuparia*, symptoms were found on inoculated seedlings as well as on controls. However, 64% (14 of 22 plants) of the inoculated individuals exhibited mild disease symptoms already at the date of inoculation. Thus, eleven seedlings were rated with a DS ranging from 0.25 to 0.75, whereas another three seedlings even showed a DS between 1 and 1.5 at that time, resulting in a MDS of 0.39. MDS increased steadily over the period of the experiment and finally reached 1.51 at the end of the vegetation period (10 WPI) (Fig. 2). At that date, twelve of 22 plants (55%) exhibited a DS ≥ 1, whereof three seedlings (14%) had a rating of 4 (i.e. were completely defoliated or dead) and another individual had a DS value of 3.75 (Table 1). Symptomatic *S*. *aucuparia* seedlings showed slight to severe necrosis on leaves and in some cases also wilting as well as dieback of buds, shoot tips, of the upper shoots or even of the whole plant. At the same time, however, new shoots emerged on some seedlings. The controls showed a partially parallel but clearly lower MDS curve: at the beginning of the experiment the controls did not show any symptoms, which changed 4 WPI (MDS 0.19) and at the of the vegetation period (10 WPI) MDS was 0.25 (Fig. 2). At that time, 50% (2 of 4 plants) of the control seedlings had developed leaf margin necrosis and suffered from aphid infestation. Surprisingly, none of the inoculated plants showed aphid infestation, although they were standing next to the control group. Statistical analyses revealed significantly lower AUDPC levels (p < 0.0001) of inoculated *S*. *aucuparia* seedlings compared to those of *A. altissima* and significant differences were also observed between the treated and the control group of *S*. *aucuparia*. In the repeated experiment in 2019/20 the MDS of inoculated *S*. *aucuparia* seedlings was 0.9 at the first evaluation date (13.07.2020) and 1.2 at the termination of the experiment (08.10.2020), whereas it was 0.5 and 0.4, respectively, for the controls. Disease symptoms were observed on 73% (11 of 15) of the inoculated seedlings, and 13% of the plants died, whereas values for the controls were 50% and 0%, respectively.

In the case of *U*. *glabra*, there was one inoculated plant which already had a reduced leaf mass at the date of inoculation and therefore was classified with a DS of 0.75. This particular plant exhibited further disease symptoms, which increased steadily during the experiment, finally resulting in DS value of 3.25 (10 WPI). All other seedlings of *U*. *glabra* (inoculated ones and controls) did not show any disease symptoms (MDS = 0), so curves of MDS of inoculated seedlings and controls were almost identical (Fig. 2).

### Vascular discolorations in the wood of seedlings

**Fig. 3:**
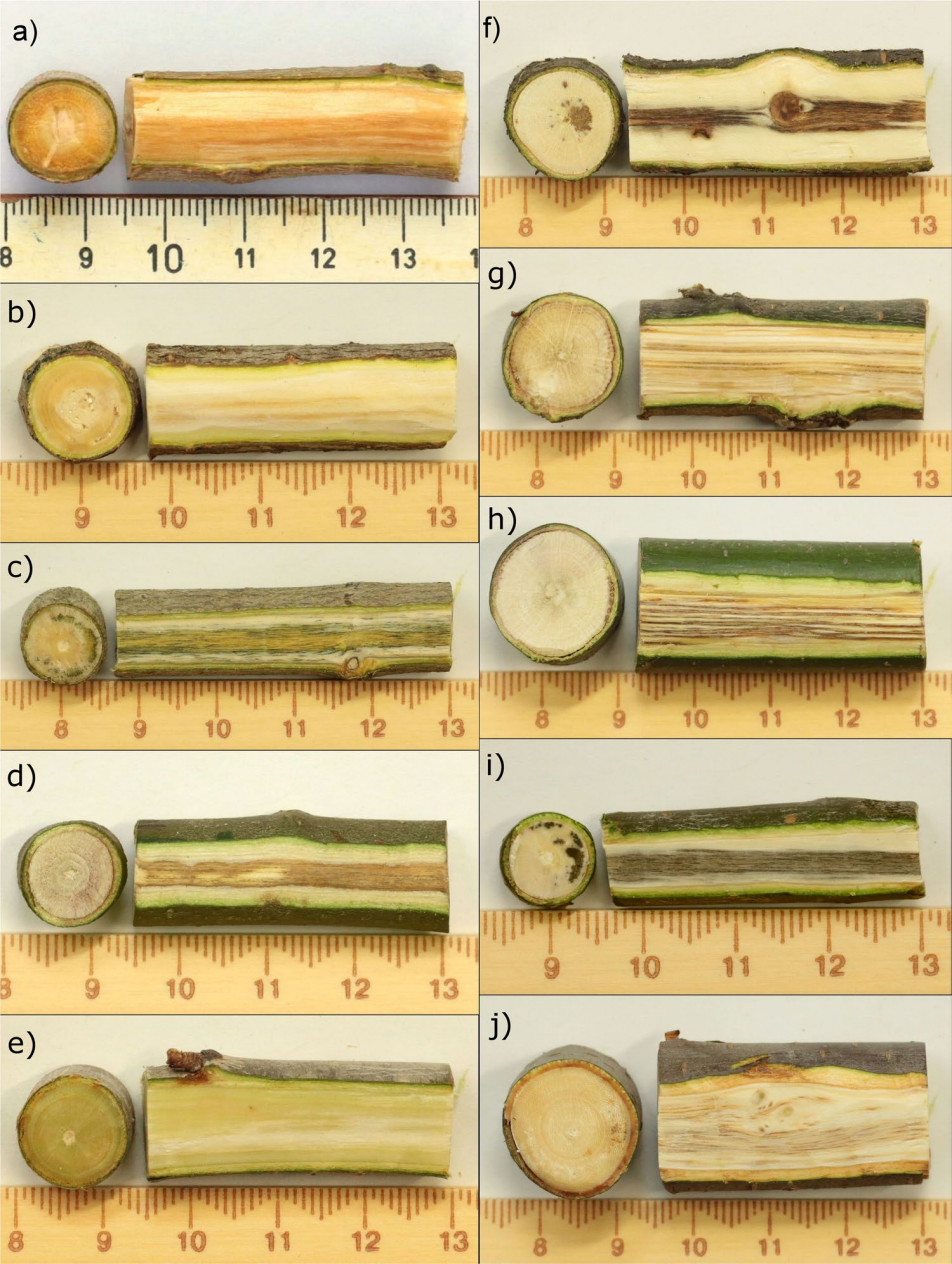
Vascular discolorations in the sapwood of inoculated seedlings of **a**) *Ailanthus altissima;* **b**) *Acer negundo*; **c**) *Acer platanoides*; **d**) *Castanea sativa*; **e**) *Prunus avium*; **f**) *Prunus serotina*; **g**) *Quercus petraea*; **h**) *Quercus rubra*; **i**) *Sorbus aucuparia*; **j**) *Ulmus glabra*

Vascular discolorations in the wood of seedlings differed in colour, intensity as well as in appearance (Fig. 3) according to the inoculated tree species. Furthermore, the number of seedlings which exhibited vascular discolorations in the sapwood varied among tree species but exceeded 60% in all tested species, except for *C*. *sativa* (Table 1)*.*

In *Ailanthus,* typical vascular discolorations in the wood were found on all inoculated seedlings: 32% of the seedlings exhibited yellowish to orange discolorations only in the early-wood vessels, 47% showed such discolorations in all current-year vessels and the remaining 21% exhibited brownish discolorations within the sapwood, which already indicated subsequent rotting.

74% (14 of 19) of the inoculated *A*. *platanoides* exhibited pronounced green to blackish-green sapwood discolorations, whereas in *C*. *sativa* orange or dark brown vascular discolorations were found in 44% (8 of 18) of the test plants. Orange to light brown discolorations developed in 91% (20 of 22) of the inoculated seedlings of *P*. *avium*, whereas inoculated seedlings of *A*. *negundo* (89%; 16 of 18) and *Q*. *rubra* (2018: 67%, 14 of 21; 2020: 100%, 15 of 15) exhibited brown sapwood discolorations. Brown to blackish sapwood discolorations were found in 62% (13 of 21) of the inoculated seedlings of *Q*. *petraea*, and in 89% (16 of 18) and 95% (21 of 22) of the examined *P*. *serotina* and *U. glabra* seedlings, respectively. *S*. *aucuparia* exhibited blackish, point-shaped vascular discolorations, which occurred in the majority of the inoculated seedlings (2018: 77%, 17 of 22; 2020: 87%, 13 of 15). However, the three (2017/18 experiment) and two (2019/20 experiment) *S*. *aucuparia* seedlings that were dead at the end of the respective vegetation periods did not show any vascular discolouration in the sapwood. In contrast, no sapwood discolorations could be found in the control seedlings.

### Re-isolations of *V*. *nonalfalfae* from seedlings

The re-isolation rate of *V. nonalfalfae* varied considerably among the examined tree species; however, there was no species, from which re-isolation of the pathogen failed completely (Table 1). Very low rates of re-isolation were obtained for *P*. *serotina* (6%) and *P*. *avium* (9%). Rates of re-isolation were moderate for *Q*. *petraea* (33%) and *A*. *negundo* (44%) and moderate to high for *Q*. *rubra* (2018: 24%; 2020: 88%). High re-isolation rates were recorded from *C*. *sativa* (61%), *U*. *glabra* (64%), *Ailanthus* (70%) and *S*. *aucuparia* (2018: 73%; 2020: 87%). With 100%, re-isolations were most successful from *A*. *platanoides*. As suspected, *V*. *nonalfalfae* could not be isolated from the controls (Table 1).

## Discussion

When using a pathogen such as *V*. *nonalfalfae* as a biological control agent against *Ailanthus*, it is of great importance to know the potential host range, in order to avoid undesirable non-target effects on associated tree species as well as negative impacts on the biocoenosis.

However, due to the taxonomic changes within the genus *Verticillium* by Inderbitzin et al. (2011), previous host range studies referring to *V*. *albo-atrum* s.l. (now comprising the species *V. albo-atrum* s.s., *V. alfalfae* and *V. nonalfalfae*) need to be treated with care or even have to be questioned. This is because it is not possible to clearly relate these studies to one of the three species, due to the lack of molecular data in these earlier papers or accessible reference cultures. Therefore, only a few reliable host range studies are currently available for *V*. *nonalfalfae*, which focused on tree species from North America (Kasson et al., 2015) and Europe (Maschek & Halmschlager, 2018). Results from Maschek and Halmschlager (2018) indicated that the *V*. *nonalfalfae* isolate Vert56 has a much narrower host range and a higher host adaption compared to *V*. *albo-atrum* s.l. Furthermore, host adaptation might even occur within *V*. *nonalfalfae* because isolate Vert56 derived from tree-of-heaven did not affect hop cultivar ‘Celeia’ (S. Radišek, pers. comm.; Dauth et al., 2022), which is known to be highly susceptible to *V. nonalfalfae* isolates obtained from hop (Radišek et al., 2006). Thus, this study further enhances our knowledge on the susceptibility of another nine tree species, often associated with *Ailanthus*, which are native, naturalized or invasive in Europe, to *V*. *nonalfalfae* isolate Vert56.

Results revealed that only *Ailanthus* seedlings inoculated with *V*. *nonalfalfae* isolate Vert56 developed characteristic external symptoms of an infection with *Verticillium* and showed a high rate of mortality, as it was already demonstrated by Maschek and Halmschlager (2018). In addition, vascular discolorations in the sapwood and the high rate of re-isolation of the pathogen confirm a high susceptibility of *Ailanthus* to this strain of *V*. *nonalfalfae*. This result is in accordance with Kasson et al. (2015), who examined the American isolate *V*. *nonalfalfae* VnAa140 and demonstrated a high virulence of that strain towards *Ailanthus*. The same was true for the American studies by Schall and Davis (2009a, b) investigating a forest with naturally infected as well as artificially inoculated *Ailanthus* trees. Severe wilting symptoms and dieback of *Ailanthus* trees after artificial inoculation were also observed on several sites in eastern Austria (Maschek, 2011; Maschek & Halmschlager, 2016a, 2017). Moreover, Moragrega et al. (2021) detected wilting and dieback on *Ailanthus* in forest ecosystems in Catalonia in Spain caused by *V*. *dahliae* and/or *V*. *nonalfalfae*. Stem inoculation experiments on *Ailanthus* with isolates obtained from these Spanish sites led to the development of disease symptoms (chlorosis, necrosis, defoliation, apical stem death and sapwood discolouration), and the pathogen could also be re-isolated from symptomatic tissues. Furthermore, O’Neal and Davis (2015a) demonstrated that *V*. *nonalfalfae* can easily spread in *Ailanthus* populations due to intraspecific root grafts, which allowed the transmission of *V*. *nonalfalfae* isolate VnAa140 from inoculated to untreated *Ailanthus* trees. Subsequently, untreated trees developed symptoms typical for *Verticillium*, and the pathogen could successfully be re-isolated from these trees. *V*. *nonalfalfae* was also found in naturalized *Ailanthus* stands in the USA (Brooks et al., 2020a; Snyder et al., 2013), where the pathogen was able to spread within these *Ailanthus* populations. The authors could observe initially healthy trees that became symptomatic over time, as well as symptomatic trees that showed dieback within the observation period.

Field studies in Europe and the USA (Maschek & Halmschlager, 2017; O’Neal & Davis, 2015b; Snyder et al., 2013) indicated that high temperatures might reduce the effectiveness of *V*. *nonalfalfae* as biological control agent or at least may slow pathogen spread (Brooks et al., 2020b). In Mediterranean areas with hot spring and summers, this problem could possibly be overcome by performing inoculations in (late) autumn, which would allow fungal infection and spread in the host within a period of the year when much cooler temperatures (compared to spring and summer) prevail. *V.* *dahliae*, which was isolated from wilting *Ailanthus* in Greece, Austria, Hungary, Italy and Spain (Skarmoutsou & Skarmoutsou, 1998; Maschek & Halmschlager, 2017; Izsépi et al., 2018; Longa et al., 2019; Pisuttu et al., 2020), is more adapted to higher temperatures (Pegg & Brady, 2002). However, this species is highly virulent to more than 200 host plants, widely distributed and very persistent in the soil due to the formation of microsclerotia, which can persist up to 14 years in the soil. Thus, considering *V.* *dahliae* as potential biological control agent against *Ailanthus* would require similar comprehensive host range studies as they have already been conducted for *V*. *nonalfalfae* (Dauth et al., 2022; Flajšman et al., 2017; Jing et al., 2018; Kasson et al., 2015; Lechner, 2019; Li et al., 2019; Maschek & Halmschlager, 2018; Schall & Davis, 2009b), with a presumed outcome that the risk of non-target effects is higher in *V.* *dahliae*.

In contrast to the high susceptibility of *Ailanthus* to *V*. *nonalfalfae*-isolate Vert56, no or only a limited impact of the pathogen was observed on the nine non-target tree species tested here: based on the development of external disease symptoms, vascular discolorations (internal symptoms) and the successful rate of re-isolation, these non-target tree species were assigned as being either tolerant or possible resistant, as it is subsequently discussed further for each of the investigated tree species.

*A*. *negundo*, which is an invasive alien species in Austria, did not develop any external symptoms, but in contrast to previously tested maple species from Europe (Maschek & Halmschlager, 2018), it was characterized by the lowest rate of re-isolation of *V*. *nonalfalfae* (*A*. *negundo*: 44%, *A*. *campestre*: 85%, *A*. *pseudoplatanus:*95% and *A*. *platanoides*: 100%). Therefore, this species was classified as possibly resistant; other *Acer* species showed a high rate of re-isolation in previous studies and were therefore classified as tolerant or even developed symptoms and were classified as susceptible (Kasson et al., 2015; Maschek & Halmschlager, 2018; Schall & Davis, 2009b). However, despite the low re-isolation rate, 89% of the tested *A*. *negundo* seedlings developed sapwood discolorations. This suggests that the pathogen may initially become established in the xylem, but is then not able to survive for a long time.

In contrast, the native *A*. *platanoides* showed the highest rate of re-isolation of *V*. *nonalfalfae* from inoculated seedlings (100%), although it also did not display external symptoms. It was therefore classified as tolerant to *V. nonalfalfae*. Similar results have been obtained by Maschek and Halmschlager (2018) for the European maple species *Acer* *campestre* and *Acer* *pseudoplatanus*, which did not exhibit external disease symptoms or mortality as well. The same was true for inoculated *Acer* *saccharum* and *A.* *rubrum* in the study by Schall and Davis (2009b). However, they could not re-isolate the pathogen from these two species. Both species were also classified as tolerant to the American isolate *V. nonalfalfae* VnAa140 by Kasson et al. (2015), as none of the inoculated test plants showed wilt, but developed sapwood discolorations and the fungus could be successfully re-isolated. Contrary results were obtained for *Acer* *palmatum*, *A*. *pensylvanicum* and *A*. *platanoides*, because these species developed typical symptoms such as wilting, defoliation, mortality and vascular discolorations and were therefore considered as susceptible (Kasson et al., 2015). *A*. *pensylvanicum* displayed the highest susceptibility, with 100% of the tested trees showing wilting and mortality. The high susceptibility of this maple species has already been described by Schall and Davis (2009b). Sinclair and Lyon (2005) classified the whole genus *Acer* as susceptible to *Verticillium* spp.; however, this classification refers to *V*. *albo*-*atrum* s.l. and *V*. *dahliae*, which were the relevant species at that time.

Comparing the results from our study with those of previous studies (Kasson et al., 2015; Maschek & Halmschlager, 2018; Schall & Davis, 2009b) for the examined *Acer* species, it appears that species within the genus *Acer* differ in their susceptibility to *V*. *nonalfalfae*. Furthermore, the contrary results obtained for *A*. *platanoides* indicate that susceptibility may even differ within one species (cf. Kasson et al., 2015 and results of this study). Observed differences might be due to differences in susceptibility of provenances of the examined tree species; however, they could also be due to differences in virulence of the applied *Verticillium* isolates from North America (*V*. *nonalfalfae* VnAa140) and Europe (*V*. *nonalfalfae* isolate Vert56).

Inoculated *C*. *sativa* did not show any symptoms in our experiment. This agrees with Sinclair and Lyon (2005), who considered the genus *Castanea* as resistant or immune to *Verticillium* spp. In contrast, 9 of 10 inoculated *Castanea* *dentata* developed wilting and died in the study of Kasson et al. (2015). However, due to a parallel infection with chestnut blight (*Cryphonectria* *parasitica*), it remained unclear whether or not the observed symptoms can be attributed to *V*. *nonalfalfae*. Incidentally, in our study some controls of *C*. *sativa* (2 of 4) developed minimal disease symptoms. As an infection with *V*. *nonalfalfae* could be excluded for the control trees, other abiotic or biotic factors were regarded to have caused these leaf margin necroses.

Six out of the 22 *P.* *avium* seedlings developed unspecific symptoms (necrosis and curling of leaves), whereas the remaining individuals stayed asymptomatic. However, five of these six seedlings already showed minor symptoms on the day of inoculation, and symptom development was restricted to seedlings that were located at the southern edge of this seedling group, where the black plant pots were directly exposed to the sun. Furthermore, curling of leaves can often be seen as a reaction of trees to avoid heat stress (Roloff, 2015). Thus, heat damage is considered as the main cause of the observed symptoms. Although all test plants were watered with the same amount of water, it also cannot be ruled out that root balls of the sun-exposed plants dried out earlier, leading, in addition, to drought stress of these seedlings. Controls of *P. avium* did not exhibit such symptoms, as they were grouped at the northern exposed edge of this seedling group; thus, plant pots were not exposed to the direct sun. In addition, an extensive undercutting of roots of the seedlings of *P. avium* was necessary for potting; thus, it can be assumed that these trees were damaged due to this procedure prior to inoculation. Apart from that, seedlings did not develop typical wilting symptoms, suggesting that the observed symptoms have not been incited by *V*. *nonalfalfae*.

In contrast to *P*. *avium*, seedlings of *P*. *serotina* did not develop any disease symptoms. Similar results were obtained by Kasson et al. (2015) for *P*. *avium* and *P*. *serotina*, where none of the tested *Prunus* plants exhibited symptoms, and these species where therefore assumed as being resistant. On the contrary, Sinclair and Lyon (2005) classified the genus *Prunus* as susceptible, but again this classification refers to *V*. *dahliae* and *V*. *albo*-*atrum* s.l. and did not differentiate between these two species.

Of all tree species tested in this study, rate of re-isolation was the lowest for both *Prunus* species (*P*. *serotina*: 6%, *P*. *avium*: 9%); at the same time, both *Prunus* species showed a high incidence of vascular discolorations in the wood (*P*. *serotina*: 89%, *P*. *avium*: 91%). This indicates that the pathogen in fact was able to colonize and possibly trigger defense reactions in the sapwood, but could hardly survive in both species until time of re-isolation. Thus, both *Prunus* species are considered largely resistant to *V*. *nonalfalfae*.

Seedlings of *Q*. *petraea* neither showed symptoms nor mortality in this study. This corresponds with the results of Maschek and Halmschlager (2018), who tested the closely related oak species *Q*. *robur*, which also did not exhibit any symptoms. The rate of re-isolation of *V*. *nonalfalfae* from inoculated seedlings of *Q*. *petraea* was substantially lower (33%) than the occurrence of vascular discolorations (62%), which suggests that the pathogen initially became established within the xylem tissues, but was then not able to survive in the host for a long time. Thus, *Q*. *petraea* is classified as possibly resistant to *V*. *nonalfalfae*.

In contrast to *Q*. *petraea*, external symptoms were observed on *Q*. *rubra* seedlings inoculated in 2017 and 2019. However, a low rate of mortality was found on seedlings inoculated in 2019 only, and observed symptoms as well as mortality could be attributed to other abiotic/biotic factors: in 2017, disease symptoms were found in both experimental groups (inoculated and control seedlings), and initial symptoms (mainly leaf margin necrosis) were already visible on the day of inoculation. Furthermore, disease progression curves of both groups were almost parallel from 6 WPI onwards. This suggests that symptom development was not related to *V*. *nonalfalfae*. In retrospect, it turned out that the quality of seedlings of this tree species was probably insufficient: observed symptoms may have arisen because bare-rooted *Q*. *rubra* seedlings, which were the tallest of all test plants, were already foliated at the day of delivery and in addition root balls had to be undercut severely for transplanting into pots. Both factors may have induced a massive drought stress on the test plants, which would fit to the observed leaf margin necrosis. In 2019, symptoms only occurred on three out of 16 inoculated *Q*. *rubra* seedlings, two of which were completely defoliated at the second evaluation date in October, whereas one seedling was already dead at that time. Due to the observed symptoms, stagnant moisture was assumed to have caused mortality of these seedlings. Since all other inoculated *Q*. *rubra* seedlings did not display any symptoms, it is very unlikely that dieback was related to *V*. *nonalfalfae*. Similar results were obtained in the study of Kasson et al. (2015), in which none out of ten inoculated *Q*. *rubra* plants wilted, but one plant died. In this case, also other abiotic or biotic factors were assumed responsible for dieback.

Re-isolations of *V*. *nonalfalfae* from *Q*. *rubra* were high in 2020 but considerably lower in 2018, which may have been because the wood was almost dead at the time of re-isolation in spring 2018 in about half of the examined *Q*. *rubra* seedlings, probably due to a very cold period (temperatures dropped up to -13 °C within a very short time) from late February until early March 2018. This makes re-isolation of a primary pathogen considerably more difficult, due to competitive effects of secondary saprobionts.

By comparing the rate of re-isolation of *V*. *nonalfalfae* from oak species tested so far, striking differences become obvious: while high rates of re-isolation (63%) were obtained for *Q*. *robur* (Maschek & Halmschlager, 2018) and *Q*. *rubra* (this study), both of which were therefore classified as tolerant, re-isolation rate was about the half or even less from *Q*. *petraea* in the present study. Thus, our results are in contrast to Kasson et al. (2015), who classified *Q*. *rubra* as resistant to *V*. *nonalfalfae* isolate VnAa140 (i.e. outwardly asymptomatic despite vascular occlusion and discolouration without successful re-isolation). In addition, results from all inoculation studies with *V*. *nonalfalfae* on oak species are in contrast to Sinclair and Lyon (2005), who classified the genus *Quercus* generally as susceptible. However, their classification again refers collectively to *V*. *dahliae* and *V.* *albo.atrum* s.l., which have a much broader host range compared to *V*. *nonalfalfae*, and does not consider the taxonomic changes made by Inderbitzin et al. (2011). This underlines the great importance of host range studies with the *Verticillium* species designated by Inderbitzin et al. (2011).

In 2017 about half, and in 2019 about three-fourths of the *Verticillium*-inoculated seedlings of *S.* *aucuparia* showed symptoms such as leaf-curling, necrosis, wilt and dieback of shoots and shoot tips and finally 14% (in 2017/18) and 13% (in 2019/20) of the plants died. However, in 2017 two-thirds of these seedlings did not look healthy already at the time of inoculation. Furthermore, also half of the seedlings from the control group exhibited similar symptoms, which finally resulted in an almost parallel progress of disease development in both groups (inoculated and control). In addition, also seedlings which have not been included/treated at all in the experiment, developed identical symptoms and some even died. In 2019, heavy infestation by aphids was found on the only three severely damaged seedlings, which may have promoted subsequent mortality. Furthermore, infestation by weevils (which caused damage due to feeding of larvae on the roots and of adults on the leaves) occurred on all symptomatic seedlings in 2019. Thus, the development of these symptoms on all plants (inoculated, control and untreated seedlings) is supposed to be the result of the inevitable undercut of root balls during transplanting of the relatively tall plants in 2017 or by the infestation of aphids and weevils in 2019. The significant reduction of root biomass in 2017 most likely also induced an adaption of crown biomass in *S.* *aucuparia*, which resulted in above-ground symptoms and poor vigor of the test plants*.* Such a close correlation between root and crown biomass has already been pointed out by Roloff (2015).

Considering the above-mentioned aspects and that successful re-isolation of *V. nonalfalfae* was achieved from 73% (in 2018) and 87% (in 2020) of the inoculated *S*. *aucuparia* seedlings, which roughly corresponds to the incidence of discolouration of the sapwood (77% in 2018 and 87% in 2020), *S*. *aucuparia* was rated as tolerant in this study. However, further inoculation trials have to be carried out to confirm this categorization. This classification is in contrast to Schröder et al. (2011) and Butin (2019), who appraised *S*. *aucuparia* as susceptible to *Verticillium*, whereas McCain et al. (1981), Pegg and Brady (2003) and Sinclair and Lyon (2005) rated the genus *Sorbus* as immune or resistant.

*V. nonalfalfae* did not incite any disease symptoms on *U. glabra.* This result is in accordance with the studies by Kasson et al. (2015) and Maschek and Halmschlager (2018), who obtained similar results for four elm species from Europe (*U. laevis, U.* *minor*), America (*U*. *americana*) and Asia (*U. pumila*) tested so far. However, due to clear differences in the rate of re-isolation, *U*. *glabra* (64%) is assigned as tolerant in the present study, whereas the European elm species tested by Maschek and Halmschlager (2018) were classified as possibly resistant, due to the low re-isolation rate of *V. nonalfalfae* (*U*. *laevis*: 10%, *U*. *minor*: 5%), and the American species *U*. *americana* and the Asian species *U*. *pumila* were assigned as resistant to the pathogen (Kasson et al., 2015).

As *V. nonalfalfae* was isolated at least at low frequencies from each of the tested tree species, no species was assigned as being resistant (R) to the pathogen in this study, whereas in the study of Maschek and Halmschlager (2018) two species (*Robinia* *pseudoacacia* and *Fraxinus* *pennsylvanica*) were classified as resistant.

Our results from artificial inoculation trials may overestimate the impact of *V*. *nonalfalfae* under field conditions. Thus, an assigned susceptibility of a tested species as a result of artificial inoculations under semi-field or controlled environmental conditions or in the greenhouse does not necessarily reflect that this species is indeed susceptible under natural field conditions (Watson, 1985; Barton, 2004). This has also been pointed out by Kasson et al. (2015), who classified *A*. *pensylvanicum* as highly susceptible in their greenhouse inoculation trials, whereas it was only moderately affected under natural conditions in declining *Ailanthus*-dominated stands. Thus, our experimental setup for host range testing very likely facilitated the growth of the pathogen, thus promoting development of disease symptoms.

Furthermore, wound inoculation of the pathogen using the method described by Maschek and Halmschlager (2016b) and applied in the present study is a “worst case” scenario, as all natural barriers of the tree aiming to prevent fungal infection are bypassed. In contrast, in the course of a natural infection, the fungus needs to actively penetrate the host tissue (Hiemstra & Harris, 1998; Pegg & Brady, 2002), which could possibly already be prevented by the presence of mycorrhiza or the exodermis (Blanchette & Biggs, 1992). Besides that, the spore concentration used in the experiments (1 × 10^7^ conidia/ml) by far exceeds naturally occurring inoculum dosages.

Moreover, the incidence of vascular discolorations and the – at least in some tree species – high rate of re-isolation from potted plants could overestimate the impact of *V*. *nonalfalfae*, as sapwood discolorations primarily confirm the successful transfer of inoculum into the xylem as well as the formation of a defense reaction of the tree (Kasson et al., 2015; Maschek & Halmschlager, 2018). Therefore, the results from artificially inoculated potted plants cannot be directly compared to the susceptibility of woody species to *V*. *nonalfalfae* under natural field conditions (Pegg & Brady, 2002).

Besides inoculation studies with potted seedlings, there are several field studies, which indicate that adjacent non-target tree species, which are listed as susceptible to *Verticillium* spp., do not develop disease symptoms in stands with naturally or artificially infected *A. altissima*. From 2011 to 2016, Maschek observed mortality of *Ailanthus* trees (naturally infected by *V*. *albo*-*atrum* s.l. and *V*. *dahliae*) in numerous forest stands in Austria, whereas all associated tree species neither developed wilt symptoms nor showed any mortality (Maschek & Halmschlager, 2017). The same was true for *A.* *campestre*, *A.* *pseudoplatanus, F. excelsior*, *R*. *pseudoacacia* and *U*. *laevis*, naturally co-occurring with artificially inoculated symptomatic *Ailanthus* in forest stands (Maschek & Halmschlager, 2018). Similar observations have been reported for *A*. *pseudoplatanus*, *C*. *sativa* and *P*. *avium* that grew intermingled with artificially inoculated *R. pseudoacacia* (Kletzmayr, 2016). The latter species also completely recovered from artificially-induced *V*. *nonalfalfae* infections.

In the USA, natural spread of *V*. *nonalfalfae* from inoculated *Ailanthus* to other tree species could be observed only in the case of two (*Aralia spinosa*, *Acer pensylvanicum*) of 38 examined species, whereas no natural spread could be recorded e.g. to *Acer* *negundo*, *A*. *platanoides*, *P.* *serotina*, *Q*. *rubra* and *Ulmus* spp. (Kasson et al., 2015). Similar results were obtained by Schall and Davis (2009b), who found associated tree species including *Q*. *rubra* and *Ulmus* spp. as tolerant to *V*. *albo*-*atrum* s.l., whereas at least low susceptibility of striped maple was observed in naturally infected stands, where 1.3% of the saplings exhibited symptoms of *Verticillium* wilt.

## Conclusions

In summary, many studies in Europe (Maschek & Halmschlager, 2016a, 2017, 2018; Moragrega et al., 2021) as well as studies from North America (Brooks et al., 2020b; Kasson et al., 2014, 2015; O’Neal & Davis, 2015b; Schall & Davis, 2009a, b) demonstrated the high potential of *V. nonalfalfae* as biological control agent against *Ailanthus*. Depending on the geographical region, different local *V. nonalfalfae* isolates have been applied so far. However, the particular host range of *V. nonalfalfae* in general as well as the specific host range of the applied isolates should largely be known, to avoid undesirable non-target effects on other species. This issue is less critical in certain areas like urban or infrastructural environments, where vegetation is undesirable anyway (e.g. along railway lines, highways, waterways, electricity line routes and at foundations of buildings), but which are often colonized by *Ailanthus.*

Results of this study and the study by Maschek and Halmschlager (2018) confirmed the high effectivity of *V*. *nonalfalfae* isolate Vert56 of strain G1/5 on invasive *Ailanthus* in Europe and indicated no or only a very limited impact on non-target tree species, as none of the 19 tree species tested so far, displayed external disease symptoms or mortality caused by the isolate after artificial inoculation. Likewise, adjacent tree species in inoculated *Ailanthus* field plots also stayed asymptomatic. As *V*. *nonalfalfae* is naturally occurring in Austria, Spain (Moragrega et al., 2021) and probably also in other European countries, it can be considered as a fairly host-specific biological control agent to combat the highly invasive tree species *A. altissima* in Europe.

## Supplementary Information

Below is the link to the electronic supplementary material.

Supplementary file1 (PDF 343 KB)

Supplementary file2 (PDF 152 KB)

## Data Availability

The datasets generated and analysed during the current study are available from the corresponding author on reasonable request.

## References

[CR1] Badalamenti E, La Mantia T (2013). Stem-injection of herbicide for control of *Ailanthus*
*altissima* (Mill.) Swingle: a practical source of power for drilling holes in stems. iForest - Biogeosciences and Forestry.

[CR2] Badalamenti E, Barone E, La Mantia T (2015). Seasonal effects on mortality rates and resprouting of stems treated with glyphosate in the invasive tree of heaven (*Ailanthus*
*altissima* (Mill.) Swingle). Arboricultural Journal.

[CR3] Barton (née Fröhlich), J. (2004). How good are we at predicting the field host-range of fungal pathogens used for classical biological control of weeds? *Biological Control, 31*(1), 99-122. 10.1016/j.biocontrol.2004.04.008

[CR4] Bejarano-Alcázar J, Blanco-López MA, Melero JM, Jiménez-Díaz RM (1996). Etiology, importance, and distribution of *Verticillium* wilt of cotton in Southern Spain. Plant Disease.

[CR5] Blanchette RA, Biggs AR (1992). Defense mechanisms of woody plants against fungi.

[CR6] Bory G, Sidibe MD, Clair-Maczulajtys D (1991). The effects of cutting back on the carbohydrate and lipid reserves in the tree of heaven (*Ailanthus*
*glandulosa* (*A.*
*altissima*)). Annales des Sciences Forestieres.

[CR7] Brooks RK, Baudoin A, Salom S (2020). The natural persistence and distribution of the proposed biological control agent *Verticillium nonalfalfae* on *Ailanthus altissima* in Virginia, USA. Forest Pathology.

[CR8] Brooks RK, Wickert KL, Baudoin A, Kasson MT, Salom S (2020). Field-inoculated *Ailanthus altissima* stands reveal the biological control potential of *Verticillium nonalfalfae* in the mid-Atlantic region of the United States. Biological Control.

[CR9] Butin H (2019). Krankheiten der Wald- und Parkbäume. Diagnose – Biologie – Bekämpfung.

[CR10] Constán-Nava S, Bonet A, Pastor E, Lledó MJ (2010). Long-term control of the invasive tree *Ailanthus altissima*: Insights from Mediterranean protected forests. Forest Ecology and Management.

[CR11] Dauth B, Maschek O, Steinkellner S, Kirisits K, Halmschlager E (2022). Non-target effects of *Verticillium nonalfalfae* isolate Vert56 used for biological control of *Ailanthus altissima* on agricultural crops known to be generally susceptible to *Verticillium* spp. Biological Control.

[CR12] Ding J, Wu Y, Zheng H, Fu W, Reardon R, Liu M (2006). Assessing potential biological control of the invasive plant, tree-of-heaven, *Ailanthus Altissima*. Biocontrol Science and Technology.

[CR13] DiTomaso JM, Kyser GB (2007). Control of *Ailanthus altissima* using stem herbicide application techniques. Arboriculture & Urban Forestry.

[CR14] Domsch KH, Gams W, Anderson TH (1980). Compendium of soil fungi.

[CR15] Drescher, A., & Magnes, M. (2002). Anthropochoren im Nationalpark Donau-Auen – Ziel von Bekämpfungsmaßnahmen oder Bereicherung der Biodiversität? BAL Bericht über das 10. Österreichisches Botanikertreffen, 30. Mai – 1. Juni 2002, 141–144. http://85.214.43.90/korina2.info/sites/default/files/Drescher%202002%20Anthropochoren.PDF. Accessed 29 Apr 2022

[CR16] European Commission. (2019). Commission Implementing Regulation (EU) 2019/1262 of 25 July 2019 amending Implementing Regulation (EU) 2016/1141 to update the list of invasive alien species of Union concern. https://eur-lex.europa.eu/legal-content/EN/TXT/PDF/?uri=CELEX:32019R1262&from=EN. Accessed 2 Dec 2021

[CR17] Filippou P, Bouchagier P, Skotti E, Fotopoulos V (2014). Proline and reactive oxygen/nitrogen species metabolism is involved in the tolerant response of the invasive plant species *Ailanthus altissima* to drought and salinity. Environmental and Experimental Botany.

[CR18] Flajšman, M., Radišek, S., & Javornik, B. (2017). Pathogenicity assay of *Verticillium nonalfalfae* on hop plants. *Bio-protocol, 7*(6). 10.21769/BioProtoc.2171. 10.21769/BioProtoc.2171 PMC8376619 34458482

[CR19] Gómez-Aparicio L, Canham CD (2008). Neighbourhood analyses of the allelopathic effects of the invasive tree *Ailanthus altissima* in temperate forests. Journal of Ecology.

[CR20] Gutte P, Klotz S, Lahr C, Trefflich A (1987). *Ailanthus altissima* (MILL.) SWINGLE – eine vergleichend pflanzengeographische Studie. Folia Geobotanica et Phytotaxonomica.

[CR21] Halmschlager E, Maschek O (2019). Biologische Kontrolle des Götterbaums. AFZ-DerWald.

[CR22] Heisey RM (1997). Allelopathy and the secret life of *Ailanthus altissima*. Arnoldia.

[CR23] Hiemstra JA, Harris DC (1998). A compendium of Verticillium wilts in tree species.

[CR24] Hu SY (1979). Ailanthus. Arnoldia.

[CR25] Inderbitzin P, Subbarao KV (2014). *Verticillium* Systematics and Evolution: How confusion impedes Verticillium Wilt management and how to resolve it. Phytopathology.

[CR26] Inderbitzin P, Bostock RM, Davis RM, Usami T, Platt HW, Subbarao KV (2011). Phylogenetics and taxonomy of the fungal vascular wilt pathogen *Verticillium*, with the descriptions of five new species. PLoS ONE.

[CR27] Izsépi F, Varjas V, Tóth T, Koncz L, Tenorio-Baigorria I, Végh A (2018). First Report of Verticillium Wilt of *Ailanthus altissima* in Hungary caused by *Verticillium dahliae*. Plant Disease.

[CR28] Jeger MJ, Viljanen-Rollinson SLH (2001). Use of the area under the disease-progress curve (AUDPC) to assess quantitative disease resistance in crop cultivars. Theoretical and Applied Genetics.

[CR29] Jing R, Li H, Hu X, Shang W, Shen R, Guo C, Guo Q, Subbarao KV (2018). Verticillium wilt caused by *Verticillium*
*dahliae* and *V.*
*nonalfalfae* in Potato in Northern China. Plant Disease.

[CR30] Kasson MT, Short DPG, O’Neal ES, Subbarao KV, Davis DD (2014). Comparative pathogenicity, biocontrol efficacy, and multilocus sequence typing of *Verticillium nonalfalfae* from the invasive *Ailanthus altissima* and other hosts. Phytopathology.

[CR31] Kasson MT, O’Neal ES, Davis DD (2015). Expanded host range testing for *Verticillium nonalfalfae*: Potential biocontrol agent against the invasive *Ailanthus altissima*. Plant Disease.

[CR32] Kleinbauer I, Dullinger S, Klingenstein F, May R, Nehring S, Essl F (2010). Ausbreitungspotenzial ausgewählter neophytischer Gefäßpflanzen unter Klimawandel in Deutschland und Österreich.

[CR33] Kletzmayr, K. (2016). Vorstudie zur biologischen Bekämpfung von *Robinia pseudoacacia* (Robinie). Master Thesis, University of Natural Resources and Life Sciences, Vienna.

[CR34] Kowarik I, Böcker R (1984). Zur Verbreitung, Vergesellschaftung und Einbürgerung des Götterbaumes (*Ailanthus altissima* [Mill.] Swingle) in Mitteleuropa. Tuexenia.

[CR35] Kowarik I, Säumel I (2007). Biological flora of Central Europe: *Ailanthus altissima* (Mill.) Swingle. Perspectives in Plant Ecology, Evolution and Systematics.

[CR36] Kowarik I (2010). Biologische Invasionen: Neophyten und Neozoen in Mitteleuropa.

[CR37] Kowarik I, Säumel I (2014). Enzyklopädie der Holzgewächse: Handbuch und Atlas der Dendrologie.

[CR38] Lechner, Y. (2019). Ergänzende Versuche zur Wirksamkeit von *Verticillium nonalfalfa*e an *Ailanthus altissima* und vergesellschafteten heimischen bzw. invasiven Baumarten in Ostösterreich. Master Thesis. University of Natural Resources and Life Sciences, Vienna.

[CR39] Lewis K, McCarthy B (2008). Nontarget Tree Mortality after Tree-of-Heaven (*Ailanthus altissima*) Injection with Imazapyr. Northern Journal of Applied Forestry.

[CR40] Li H, Wang Z, Hu X, Shang W, Shen R, Guo Ch, Guo Q, Subbarao KV (2019). Assessment of resistance in potato cultivars to verticillium wilt caused by *Verticillium*
*dahliae* and *Verticillium*
*nonalfalfae*. Plant Disease.

[CR41] Ließ, N. (2007). Der Baum des Himmels? – *Ailanthus altissima* (Mill.) Swingle. Monitoring und Evaluierung von Kontrollmethoden im Nationalpark Donau-Auen (Österreich). Master Thesis. University of Applied Sciences for Sustainable Development.

[CR42] Ließ N, Drescher A (2008). *Ailanthus altissima* spreading in the Danube National Park – possibilities of control. Neobiota.

[CR43] Lin L, Peiser G, Ying B, Mathias K, Karasina F, Wang Z, Itatani J, Green L, Hwang Y (1995). Identification of plant growth inhibitory principles in *Ailanthus*
*altissima* and *Castela*
*tortuosa*. Journal of Agricultural and Food Chemistry.

[CR44] Longa CMO, Pietrogiovanna M, Minerbi S, Andriolo A, Tolotti G, Maresi G (2019). First observation of verticillium wilt on *Ailanthus altissima* in the Eastern Italian Alps (Trentino-South Tyrol). Journal of Plant Pathology.

[CR45] Maschek, O. (2011). Untersuchungen zur biologischen Bekämpfung von *Ailanthus altissima.* Master Thesis, University of Natural Resources and Life Sciences, Vienna.

[CR46] Maschek O, Halmschlager E (2016). First report of *Verticillium* wilt on *Ailanthus altissima* in Europe caused by *Verticillium nonalfalfae*. Plant Disease.

[CR47] Maschek O, Halmschlager E (2016). A rapid, reliable and less-destructive method for stem inoculations on trees. Forest Pathology.

[CR48] Maschek O, Halmschlager E (2017). Natural distribution of *Verticillium* wilt on invasive *Ailanthus altissima* in eastern Austria and its potential for biocontrol. Forest Pathology.

[CR49] Maschek O, Halmschlager E (2018). Effects of *Verticillium nonalfalfae* on *Ailanthus altissima* and associated indigenous and invasive tree species in eastern Austria. European Journal of Forest Research.

[CR50] McCain, A. H., Raabe, R. D., & Wilhelm, S. (1981). Plants Resistant to or Susceptible to *Verticillium* Wilt. University of California Leaflet 2703.

[CR51] Moragrega, C., Carol, J., Bisbe, E., Fabregas, E., & Llorente, I. (2021). First report of *Verticillium* wilt and mortality of *Ailanthus altissima* caused by *Verticillium dahliae* and *V*. *albo*-*atrum* sensu lato in Spain. *Plant Disease, 105*(11). 10.1094/PDIS-03-21-0463-PDN

[CR52] Müller, R. (2012). Evaluierung von Bekämpfungsmaßnahmen gegen den Götterbaum (*Ailanthus altissima*) im Nationalpark Donau-Auen (Österreich). Bachelor Thesis, Dresden University of Technology.

[CR53] O’Neal ES, Davis DD (2015). Intraspecific root grafts and clonal growth within *Ailanthus altissima* stands influence *Verticillium nonalfalfae* transmission. Plant Disease.

[CR54] O’Neal ES, Davis DD (2015). Biocontrol of *Ailanthus altissima*: Inoculation protocol and risk assessment for *Verticillium nonalfalfae* (Plectosphaerellaceae: Phyllachorales). Biocontrol Science and Technology.

[CR55] Pegg GF, Brady BL (2002). Verticillium wilts.

[CR56] Pisuttu C, Marchica A, Bernardi R, Calzone A, Cotrozzi L, Nali C, Pellegrini E, Lorenzini G (2020). Verticillium wilt of *Ailanthus*
*altissima* in Italy caused by *V.*
*dahliae*: new outbreaks from Tuscany. iForest.

[CR57] Punz W, Aigner B, Schimpl C, Pietsch G, Schosmeier E, Maier R (1998). Stadtbrachen in Wien. Verhandlungen der zoologisch-botanischen Gesellschaft Wien.

[CR58] Radišek S, Jakše J, Javornik B (2006). Genetic variability and virulence among *Verticillium albo-atrum* isolates from hop. European Journal of Plant Pathology.

[CR59] Rebbeck, J., Malone, M. A., Short, D. P. G., Kasson, M. T., O’Neal, E. S., & Davis, D. D. (2013). First report of *Verticillium* wilt caused by *Verticillium nonalfalfae* on Tree-of-Heaven (*Ailanthus altissima*) in Ohio. *Plant Disease, 97*(7). 10.1094/PDIS-01-13-0062-PDN 10.1094/PDIS-01-13-0062-PDN 30722582

[CR60] Roloff A (2015). Handbuch Baumdiagnostik: Baum-Körpersprache und Baum-Beurteilung.

[CR61] Schall MJ, Davis DD (2009). *Ailanthus altissima* wilt and mortality: Etiology. Plant Disease.

[CR62] Schall MJ, Davis DD (2009). *Verticillium* wilt of *Ailanthus altissima*: Susceptibility of associated tree species. Plant Disease.

[CR63] Schröder T, Bräsicke N, Schuhmacher J, Wulf A (2011). Schadorganismen an der Elsbeere. Krankheiten und Schädlinge am Baum des Jahres 2011. AFZ-Der Wald.

[CR64] Siegrist M, Holdenrieder O (2016). Die *Verticillium*-Welke – eine Option zur Bekämpfung des Götterbaumes in der Schweiz?. Schweizerische Zeitschrift für Forstwesen.

[CR65] Sinclair WA, Lyon HH (2005). Diseases of trees and shrubs.

[CR66] Skarmoutsou G, Skarmoutsou H (1998). Occurrence of wilt disease caused by *Verticillium*
*dahliae* on *Ailanthus*
*glandulosa* in Greece. Plant Disease.

[CR67] Snyder AL, Salom SM, Kok LT (2013). Survey of *Verticillium nonalfalfae* (Phyllachorales) on tree-of-heaven in the southeastern USA. Biocontrol Science and Technology.

[CR68] Svara A, Jakše J, Radišek S, Javornik B, Stajner N (2019). Temporal and spatial assessment of defence responses in resistant and susceptible hop cultivars during infection with *Verticillium nonalfalfae*. Journal of Plant Physiology.

[CR69] Watson, A. K. (1985). Host specificity of plant pathogens in biological weed control. Proc. VI Int Symp Biol Control Weeds, Vancouver, BC, Canada: 577–586.

[CR70] Wickert KL, O’Neal ES, Davis DD, Kasson MT (2017). Seed production, viability, and reproductive limits of the invasive *Ailanthus altissima* (Tree-of-Heaven) within invaded environments. Forests.

