# Supplementary material for: Further pathogenicity testing of Verticillium nonalfalfae, a biocontrol agent against the invasive Tree of Heaven (Ailanthus altissima), on non-target tree species in Europe
Source: Phytoparasitica. 2022 Nov 5;51(1):113–30. doi: 10.1007/s12600-022-01032-z (PMC9638367; doi:10.1007/s12600-022-01032-z)
Supplement: Supplementary file 1 — Supplementary file1 (PDF 343 KB) [file 12600_2022_1032_MOESM1_ESM.pdf]

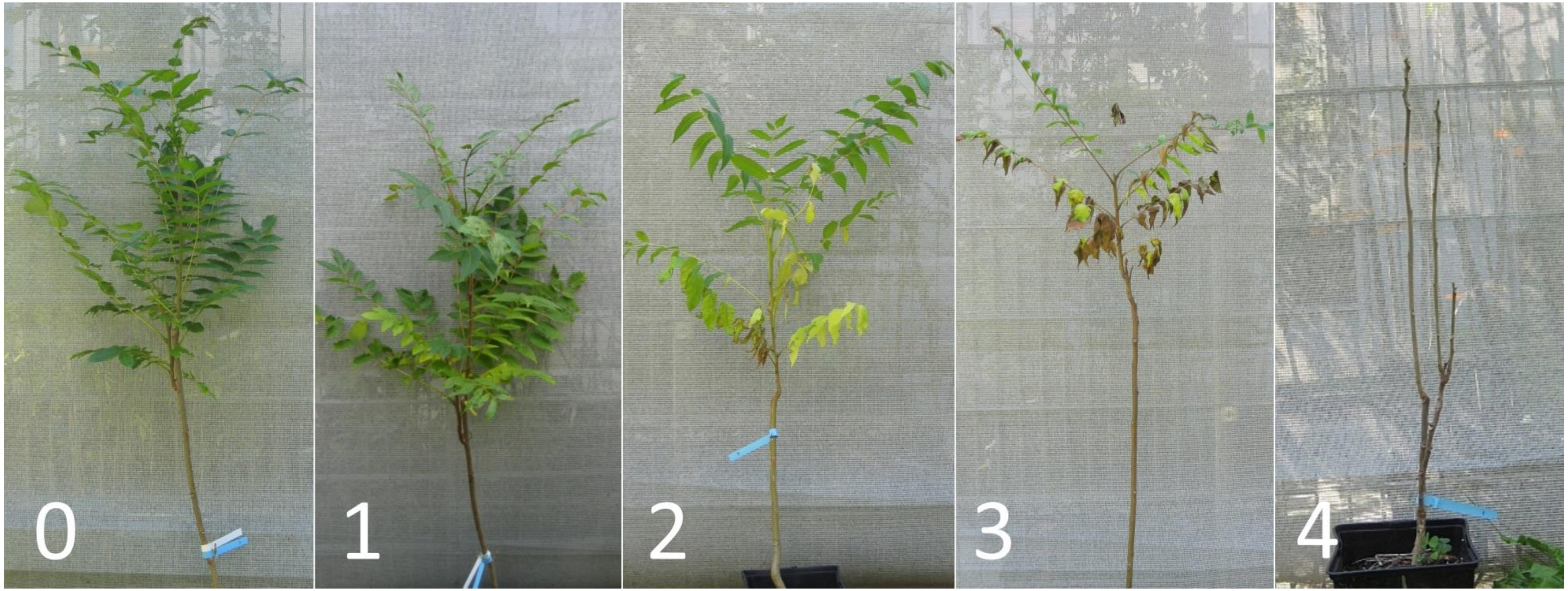


**Fig. S1** Series of photographs reflecting the different categories (0 to 4) of disease severity (DS) examined on inoculated seedlings of *Ailanthus altissima*
