# Supplementary material for: Further pathogenicity testing of Verticillium nonalfalfae, a biocontrol agent against the invasive Tree of Heaven (Ailanthus altissima), on non-target tree species in Europe
Source: Phytoparasitica. 2022 Nov 5;51(1):113–30. doi: 10.1007/s12600-022-01032-z (PMC9638367; doi:10.1007/s12600-022-01032-z)
Supplement: Supplementary file 2 — Supplementary file2 (PDF 152 KB) [file 12600_2022_1032_MOESM2_ESM.pdf]

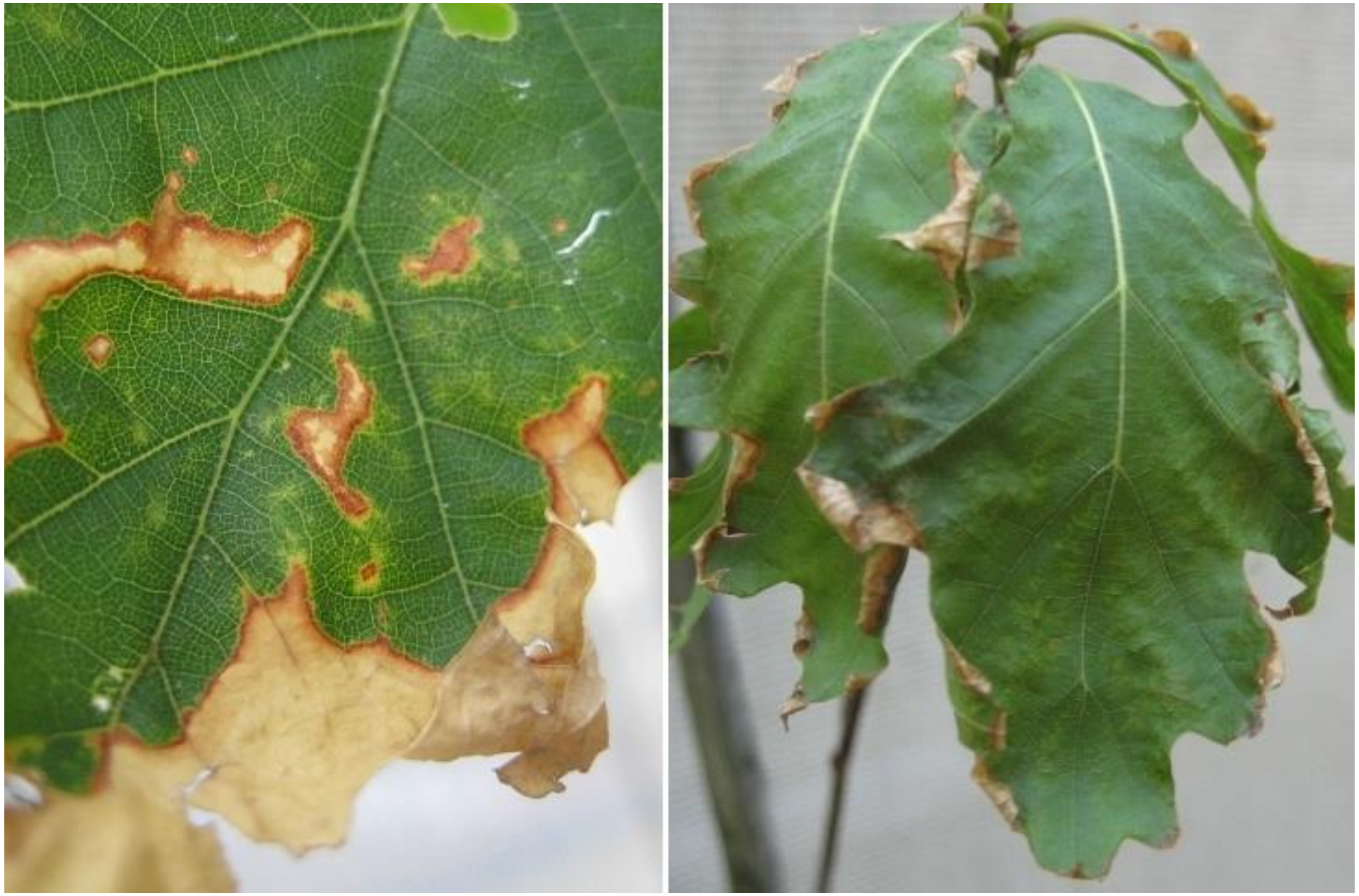

Fig. S2 Leaf margin necroses observed on *V. nonalfalfae*-inoculated *Quercus rubra* seedlings (left, 8 WPI) but also on controls (right, 6 WPI) that were attributed to radical root cut during potting
